# Detecting Horizontal Gene Transfer between Closely Related Taxa

**DOI:** 10.1371/journal.pcbi.1004408

**Published:** 2015-10-06

**Authors:** Orit Adato, Noga Ninyo, Uri Gophna, Sagi Snir

**Affiliations:** 1 Department of Evolutionary Biology, University of Haifa, Haifa, Israel; 2 Department of Molecular Microbiology and Biotechnology Tel Aviv University, Tel-Aviv, Israel; The Centre for Research and Technology Hellas, Greece

## Abstract

Horizontal gene transfer (HGT), the transfer of genetic material between organisms, is crucial for genetic innovation and the evolution of genome architecture. Existing HGT detection algorithms rely on a strong phylogenetic signal distinguishing the transferred sequence from ancestral (vertically derived) genes in its recipient genome. Detecting HGT between closely related species or strains is challenging, as the phylogenetic signal is usually weak and the nucleotide composition is normally nearly identical. Nevertheless, there is a great importance in detecting HGT between congeneric species or strains, especially in clinical microbiology, where understanding the emergence of new virulent and drug-resistant strains is crucial, and often time-sensitive.

We developed a novel, self-contained technique named *Near HGT*, based on the *synteny index*, to measure the divergence of a gene from its native genomic environment and used it to identify candidate HGT events between closely related strains. The method confirms candidate transferred genes based on the *constant relative mutability* (CRM). Using CRM, the algorithm assigns a confidence score based on “unusual” sequence divergence. A gene exhibiting exceptional deviations according to both synteny and mutability criteria, is considered a validated HGT product. We first employed the technique to a set of three *E. coli* strains and detected several highly probable horizontally acquired genes. We then compared the method to existing HGT detection tools using a larger strain data set.

When combined with additional approaches our new algorithm provides richer picture and brings us closer to the goal of detecting all newly acquired genes in a particular strain.

## Author Summary

The transfer of genetic material between organisms, usually denoted as horizontal (or lateral) gene transfer (HGT or LGT), is a prime mechanism in microbial evolution and responsible for genetic innovation and the evolution of genome architecture. Detecting HGT between closely related species or strains is imperative as drug-resistant pathogenic strains most often acquire their virulence from closely related bacteria. The proposed method combines two evolutionary signals that were not employed in the past for this task. One is the synteny index (SI), measuring the loss of synteny in an organism, and the other is a novel concept—constant relative mutability (CRM), maintaining that genes preserve their relative evolution rate along linages (although the latter ones may each change).

We show both in simulation and real biological data that the method is sound and, in the cases examined, provides stronger sensitivity than existing methods. We therefore believe this novel approach represents a significant advance, for the first time enabling the detection of previously ignored HGT events that will bring us closer to the goal of detecting all newly acquired genes in a particular strain.

**Availability:** The method is publicly available at http://research.haifa.ac.il/~ssagi/software/nearHGT.zip

This is a *PLOS Computational Biology* Methods paper.

## Introduction

**Fig 1:**
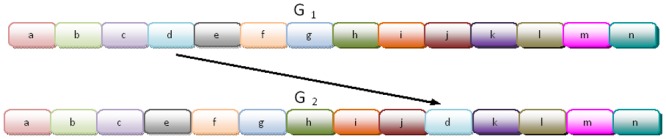
Gene *d* was transferred from donor species *G*_1_ to recipient species *G*_2_.

Most microbial genomes have experienced extensive gene mobility between lineages during their evolution, a phenomenon known as horizontal gene transfer (HGT). This process has been critical in shaping microbial genome evolution both in terms of functional repertoires and of genome architecture [1, 2, 3, 4, 5, 6]. Many HGT events result in a gene being copied from the donor genome to the recipient genome (see Fig 1), and this process can be mediated by integration of viruses (bacteriophages), transposable elements, or integrative plasmids, often by non-homolgous recombination.

The study of the HGT is of paramount importance for several reasons. First, from a clinical perspective, HGT plays a major role in the emergence of new human diseases, as well as promoting the spread of antibiotic resistance in bacterial species [7, 8]. From the fundamental, evolutionary standpoint, HGT links distant branches in the tree of life, turning it into an evolutionary network [9, 3, 10]. Genetically, HGT is an important, if not the primary, source of genetic novelty by bacteria and archaea and often results in adaptations to new environments and conditions [11]. Recent advances of comparative genomics and especially metagenomics indicate that the complexity of the genetic material that is horizontally transferred, is vast and often exceeds by orders of magnitude the complexity of the set of conserved genes that are mostly vertically inherited [12]. Therefore, correct identification of HGT can shed light on many significant evolutionary processes some of which are adaptive.

Currently, there are two prevailing methods for detecting HGT. The *phylogeny based approach* takes a relatively large set of copies of the investigated gene (may contain several copies at a species due to duplication), constructs their corresponding phylogeny and contrasts it to the phylogeny of their originating species. When conflicts are found between the two trees, they are reconciled by introducing HGTs or other events (see e.g. [13, 14, 15, 16, 17]). While this approach has the advantage of identifying relatively ancient events, it is based on a very stringent assumption of where to seek the events—which is the transferred gene. Additionally, it also requires a *multiple sequence alignment* (MSA) of the sequences, and inferring a reliable species tree (two major problems by themselves [18, 19], in particular where phylogenetic signal is weak). In contrast, the *composition based approach* contrasts genomic sequences of different compositional features such as G+C content, dinucleotide frequencies or codon usage biases, striving to detect genes with different origins than the rest of the genome (e.g. [20, 5, 6, 21, 22]). The latter approach suffers from the fact that the species involved might share similar compositional patterns. Moreover, the length of a transferred segment may be too short to reliably reveal these differences. As concluded in [23, 24], “atypical G+C content and pattern of codon usage are not reliable indicators of horizontal gene transfer events”.

Both the phylogenetic and the sequence composition based approaches must rely on strong enough signals for detecting HGT: The phylogenetic approach requires the transferred gene to be relatively distinct from its close relatives’ counterparts and at the same time resemble a relatively distant species in the taxa set [14, 25]. The sequence composition based approach, requires the transferred segment to be of relatively distant origin, so that enough divergence has accumulated to result in different compositional features. Thus, to maximize sensitivity and accuracy HGT detection should use an array of approaches to either detect new events or confirm events detected by one method, using rival methods [26].

The discussion above raises the problem of detecting HGT between closely related species or even strains of the same species, where a strong enough signal for existing HGT detection methods may not exist. This calls for a new distinction of *intra-clade* HGT in which both donor and recipient organisms are from the same broadly defined lineage, and *inter-clade* HGT where the donor and recipient are from different, and distant lineages. Such a solution is required when exploring the sudden emergence of drug-resistant pathogenic strains, which most often acquire their virulence from closely related bacteria. In this work we make a first step in this direction and present a novel technique for detecting HGT between closely related species or strains, that we refer to as intra-clade HGT. The technique builds on the concept of—the *synteny index* (SI) between two genomes (species) that we previously developed [27].

Gene synteny [28, 29] is the conservation of gene order across species along the evolutionary course. Synteny (or lack of) was already employed for defining a distance measure between genomes (species). Under this formulation, two genomes over the same set of genes are viewed as a permutation of one another and the task is to find the minimal number of legal operations to transform one genome to another [30, 31, 32]. Nevertheless the rearrangement distance is irrelevant in the context of a particular gene and therefore cannot be used to detect HGT. In contrast, SI measures how much a gene, orthologous to the two species, is in its “natural place”, or in other words, shares the same neighborhood in both genomes. The two underlying assumptions are that a newly acquired gene is inserted at a random location and therefore with high probability in a new neighborhood and that, closely related species have undergone low level of HGT activity (since they are closely related). We also define the *average SI* between two genomes that is a weighted average of SI’s and extends the SI from the gene-level to the genome-level. Average SI provides a measure of divergence in a population exposed to frequent HGT activity. Since low average SI is indicative of high divergence (and vice verse for high average SI) [33, 27], we can exploit a gene-specific low SI between closely related species (that exhibit high average SI), to detect potential HGTs for that gene. Hence, the core set of genes shared by two organisms, can be a basis to generate the SI distribution between them where genes of exceptionally low SI are marked as *SI HGT candidates*. As low SI can be a result of other global genomic rearrangements [34], we need to account for these events (see in Fig 1, genome *G*_2_ can equally be resulted by a translocation of gene *d* from between *c* and *e* to between *j* and *k*). Here we rely on the *constant relative mutability (CRM)* property that is a direct product of the *Universal Pacemaker (UPM) of genome evolution* [35, 36] phenomenon. This property asserts that, in general, and across all lineages of the tree of life, any two genes preserve the same ratio between their respective evolutionary rates. In particular, this measure was tested and validated in bacteria [35, 37], the organisms we analyze here. Using this property, we can calculate the expected distance between the two copies of a gene that SI has indicated to be a HGT candidate in the studied organisms. Using a statistical confidence check, a reinforcement for the HGT hypothesis is obtained. We applied our method to real biological data, the three strains of *E. coli* that were studied in [38] and were found to exhibit a very high rate of HGT. Understanding and detecting HGT within the strains, could be of great importance, for instance in understanding the origin of pathogenicity of certain pathogenic strains, particularly those whose ancestors were not pathogenic. While [38] focused on inter-HGT among these species by means of codon usage, they could not detect intra-HGTs between the strains themselves. Our method detected several genes with high probability of being horizontally transferred. For a sample of them, we checked for HGT by other complementary methods, such as RIATA-HGT [39] and PhylTr [40], and obtained supporting evidence for our inferences. These results suggest a combined approach in which the lightweight approach Near HGT is first used to detect putative HGTs where the signal is weak (e.g. among strains). Next heavier approaches such as the phylogenetic approaches, are used where the signal is more pronounced or to confirm putative specific events first found by Near HGT.

The method with an accompanying documentation and examples, along with the procedures used for this study is available at http://research.haifa.ac.il/~ssagi/software/nearHGT.zip. Supplementary material used in this study is available at http://research.haifa.ac.il/~ssagi/SI-HGT/suppl.zip

## Results

In this section we describe our novel algorithm, ***Near HGT*** for detecting putative HGTs between closely related species, and subsequently, results from applying it on a set of *E. coli* strains.

### Near HGT—Detecting Horizontal Gene Transfer between Closely Related Organisms

Since SI is defined for a single specific gene shared by two genomes, we can exploit that property for gene specific studies. As demonstrated in [27], closely related species exhibit high average SI reflecting the fact that their respective genes normally share the same neighborhood. Our underlying assumption is that an acquired gene is inserted in a random location. Hence, between closely related species (and in particular strains of a species), if a gene has exceptionally low SI, we might suspect it has undergone HGT. Indeed looking at the histogram of SI between three strains of *E. coli*: CFT073, EDL933 and MG1655 in section [Analysis of Real Biological Data] below, reveals very high gene counts at the high SI values (bars at the right end corresponding to *SI* ∈ [17, 20]) and very low gene counts for the low SI, *SI* ∈ [1, 5]. The absolute values for these SI distributions can be found at table in S2 Table in the supplementary material. A notable rise is found for *SI* = 0. We suspect this reflects genes acquired by HGT. Therefore, given some *threshold SI value* 0 < *δ*_*SI*_ < 1, we define an *SI cutoff* *C*(*δ*_*SI*_), such that the fraction of genes *g*_0_ for genomes *G*_*i*_, *G*_*j*_, *SI*(*g*_0_, *G*_*i*_, *G*_*j*_) ≤ *C*(*δ*_*SI*_), is less then *δ*_*SI*_. We denote these genes as *SI HGT suspected*. We note though, that by low SI we cannot distinct between donor and recipient. Moreover low SI is exhibited between the recipient and generally every other genome. Therefore, as we indicate in our real data analysis, when multiple genomes are analyzed, a clearer view is provided.

Next it is important to verify that these genes are indeed the result of a HGT event. This is important as low SI can also be a product of other large scale genomic events: a *translocation*, an event where a gene moves to a different location in a genome, or a *Duplication*, a similar event where a copy of the gene remains in the original location.

The following observation follows intuitively from Fig 1.

**Observation 0.1.** *Let G*_1_ *and G*_2_ *be two genomes sharing a common gene g. Assume g was either translocated or duplicated in G_2_ (we assume g corresponds to the copied instance rather than the original). Assuming no other large scale genomic events occurred, then with high probability SI*(*g, G*_1_, *G*_2_) = 0.

Indeed, based on SI only, it cannot be distinguished whether a gene has been horizontally transferred or simply translocated within the genome. Therefore we cannot rely on low SI as the sole evidence for HGT. To establish that a gene has undergone HGT we rely on the fact that a translocated (duplicated) gene has resided in its host genome a sufficiently long time since its split from another genome (one belonging to another strain or species), in contrast to a gene recently acquired through HGT. This implies that the translocated gene was subjected to small scale substitutions (such as point mutations) for the time period since its split from the other genome. Hence the inferred distance between orthologous genes in two genomes, is proportional to the time since their divergence.

Therefore, to distinguish an HGT from translocations or duplications, we rely on the fact that a translocated (duplicated) gene has been in its hosting genome since its split from another genome, in contrast to a gene recently acquired through HGT.

We now rely on a very basic evolutionary effect recently demonstrated, dubbed as *Universal Pacemaker (UPM) of genome evolution* [35, 36]. The UPM principle states that along every lineage in the evolution of cellular life, most genes change their mutation rate in unison, as if adhering to a universal (but lineage specific) pacemaker.

We now observe the basic property, denoted as *constant relative mutability* (CRM), which we exploit in this part and is a direct outcome of the UPM: For every two genes *g* and *g*′ residing in a genome *G* mutating at (not necessarily constant) rates *α* and *α*′, the ratio *ρ*_*g*, *g*′_ = *α*/*α*′ is (approximately) constant at all times.

The CRM property can be utilized for our task in the following way. If a gene *g*_*h*_ has undergone a HGT between two species *s*_1_ and *s*_2_, then the evolutionary distance between these very species according to this gene *g*_*h*_ has shortened, proportionally to the time of the HGT event. However, since the HGT is unknown, this short distance between *s*_1_ and *s*_2_ according to *g*_*h*_ cannot be attributed with certainty to a HGT event, but rather to conservation of *g*_*h*_, or to the case that *g*_*h*_ has slowed its rate along these specific lineages (recall that the evolutionary tree is not known and in particular, this tree according to *g*_*h*_ is substantially jumbled). Now, the CRM property comes to play. It manifests that regardless of the characteristic rate of *g*_*h*_, and even if it slowed down, it maintains (relatively) the same ratio to all other gene rates along that lineage. Therefore, the following is done: An additional *witness* gene *g*_*w*_, and two additional *reference organisms* *r*_1_ and *r*_2_ are taken arbitrarily and assume the time separating between *r*_1_ and *r*_2_ is *t*(*r*_1_, *r*_2_). Now, the rate ratio between *g*_*h*_ and *g*_*w*_, *ρ*_*g*_*h*_, *g*_*w*__ is calculated,

$$\rho_{g_{h},g_{w}}=\frac{d_{g_{h}}\left(r_{1},r_{2}\right)/t\left(r_{1},r_{2}\right)}{d_{g_{w}}\left(r_{1},r_{2}\right)/t\left(r_{1},r_{2}\right)}=\frac{d_{g_{h}}\left(r_{1},r_{2}\right)}{d_{g_{w}}\left(r_{1},r_{2}\right)}.$$ (1)

This is the *expected ratio* that is expected to prevail along all lineages and between any two organisms. Hence the same ratio but between *s*_1_ and *s*_2_ is now computed and this is the *observed rate ratio* $\rho_{g_{h},g_{w}}^{{}^{\prime }}$:

$$\rho_{g_{h},g_{w}}^{{}^{\prime }}=\frac{d_{g_{h}}\left(s_{1},s_{2}\right)/t\left(s_{1},s_{2}\right)}{d_{g_{w}}\left(s_{1},s_{2}\right)/t\left(s_{1},s_{2}\right)}=\frac{d_{g_{h}}\left(s_{1},s_{2}\right)}{d_{g_{w}}\left(s_{1},s_{2}\right)}.$$ (2)

Now, by the CRM hypothesis, $\rho_{g_{h},g_{w}}^{{}^{\prime }}=\rho_{g_{h},g_{w}}$ and this is indeed our null hypothesis. As we suspect the “rate” of *g*_*h*_ has changed as a result of HGT (we use quotation marks as the rate of *g*_*h*_ has not really changed, but rather the time of divergence is different), and hence also the respective *observed distance* *d*_*g*_*h*__(*s*_1_, *s*_2_), or for short just *d*_*g*_*h*__. We now set

$$d_{g_{h}}^{\prime }=\rho_{g_{h},g_{w}}d_{g_{w}}\left(s_{1},s_{2}\right),$$ (3)

and denote it as the *expected distance* between *s*_1_ to *s*_2_ according to *g*_*h*_.

To decide whether *g*_*h*_ has undergone HGT, we use Chi-square significance test between observed and expected values [41]. In our case *d*_*g*_*h*__ and $d_{g_{h}}^{\prime }$ serve as observed and expected “coin probabilities” respectively, gene length is the coin flips, and we use degree of freedom (DoF) 1 as follows:

$$\chi^{2}=\sum_{i}\frac{{\left(O_{i}-E_{i}\right)}^{2}}{E_{i}}=\frac{{\left(\ell d_{g_{h}}-\ell d_{g_{h}}^{\prime }\right)}^{2}}{\ell d_{g_{h}}^{\prime }}+\frac{{\left(\ell \left(1-d_{g_{h}}\right)-\ell \left(1-d_{g_{h}}^{\prime }\right)\right)}^{2}}{\ell (1-d_{g_{h}}^{\prime })}$$ (4)

We refute the null hypothesis, i.e. decree if *g*_*h*_ undergone HGT, if the *χ* ^2^ probability with one degree of freedom is below another threshold value *δ*_*ρ*_.

**Fig 2:**
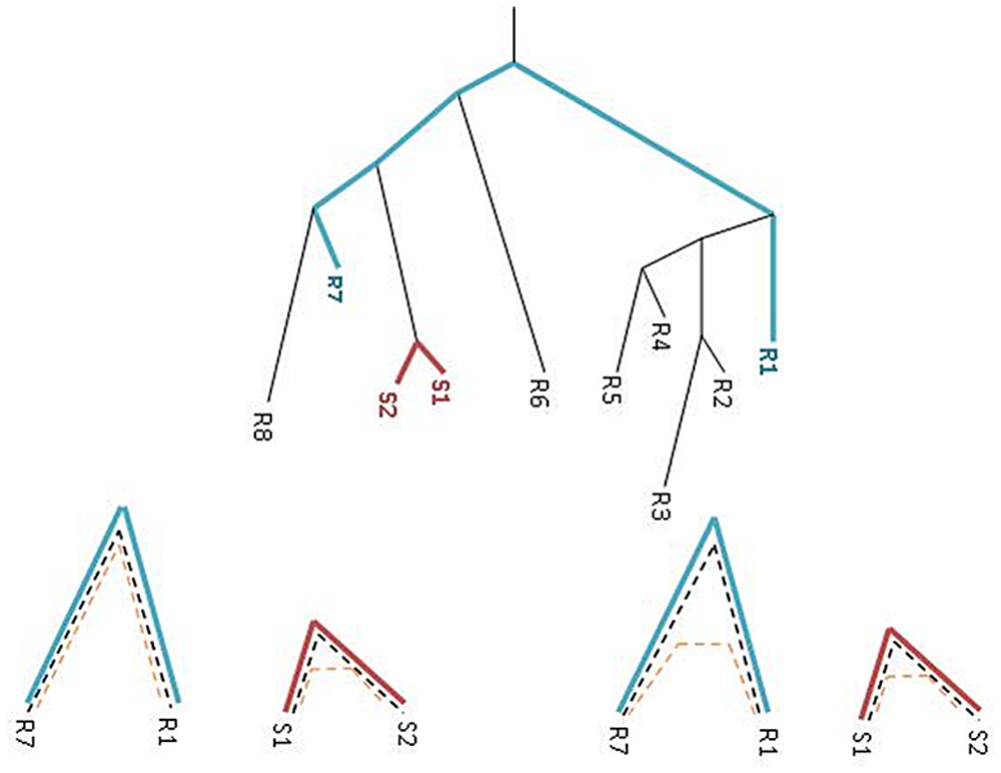
**Top:** The phylogeny over a group of organisms with branch lengths proportional to distances of gene *g*_*h*_. *g*_*h*_ has undergone HGT between the two strains *S*_1_ and *S*_2_ and hence their distance is very short compared with two reference organisms *R*_1_ and *R*_7_. **Bottom:** The reference gene (blue, dashed line) must be a gene that accumulates mutations ever since the divergence of both the strains and reference organisms. There are two cases in which the suspicious gene evolves at the reference organism. (*A*) No HGT and then the constant relative conserveness is maintained (black dashed). (*B*) HGT of the SI suspicious gene at the reference organisms and the constant relative conservation is not maintained (yellow dashed).

Fig 2 describes the situation. At the top, the tree for the reference organisms and the two strains is illustrated with proportional branch lengths. The SI-suspected gene between the two strains *S*_1_ and *S*_2_ should be compared with respect to the reference organisms. At the bottom left, HGT at the suspicious gene “shortens” the distance between the two strains, violating the constant ratio between rates (or distances).

**Example 1.** *To illustrate the use of our inference rule we show an example from our real data below. The evolutionary model with which we use is the Jukes-Cantor* [42] *(JC) evolutionary model(While we are aware it is not a realistic model, it serves here only for illustration.)*.

*Let the two strains s*_1_ *and s*_2_ *be the* E. coli *strains CFT073 and MG1655 and the reference organisms*, r_1_ *and r*_2_, *be* Bacteroides fragilis *and* Wolbachia. *The HGT suspected gene g_h_ is* engA *and the witness gene is* gmk. *We abbreviate for d_h_*(*r*) *for d_gh_*(*r*_1_, *r*_2_) *and analogously for the other cases. The distances obtained are:*

- *d*_*h*_(*s*) = 0.0080
- *d*_*w*_(*s*) = 0.0237
- *d*_*h*_(*r*) = 0.583
- *d*_*w*_(*r*) = 0.541
- *n* = 1472.

*we get:* $\rho =\frac{d_{h}(r)}{d_{w}(r)}=0.583/0.541=1.077$. *Now, by* Eq (3) *we set* $d_{g_{h}}^{\prime }=\rho d_{w}(s)=1.077*0.0237=0.0255$.

*However, we have d_gh_*(*s*) = 0.0080.

*We convert the two distances to hamming distance:* $hd_{g_{h}}(s)=(3/4)(1-e^{-(4/3)d_{g_{h}}})=(3/4)(1-e^{-(4/3)*0.0080})=0.00795$ $hd_{g_{h}}^{\prime }=(3/4)(1-e^{-(4/3)d_{g_{h}}^{\prime }})=(3/4)(1-e^{-(4/3)*0.0255})=0.02507$

*Therefore, by* Eq (4), *our* $\chi^{2}=\frac{{\left(1472(0.00795-0.02507)\right)}^{2}}{1472(0.02507)}+\frac{{\left(1472(1-0.00795)-1472(1-0.02507)\right)}^{2}}{1472(1-0.02507)}=17.65$

*Now, if we set δ_ρ_* = 0.01 *we see that* *χ* ^2^ = 17.65 *with one DoF is obtained with probability below δ_ρ_ and we can infer that the gene has undergone HGT*.

There are few cases that we can miss a gene having undergone HGT. As depicted in Fig 2 at the bottom right(marked with yellow dashed line), the SI-suspected gene might have undergone a HGT also between the reference organisms. In that case we will not detect the HGT since the rate ratio is biased in both the strains and the reference genome. It might also be that the witness gene has undergone HGT in the strains (but *not* in the reference organisms). Here as well the rate ratio is maintained and the HGT will not be detected. Finally, as the strains are evolutionarily close, for many genes, the phylogenetic signal is very weak and does not provide the distinction between HGT and vertical descent. For these reasons the complete algorithm iterates over all possible witness genes and reference organisms. Here is the complete algorithm, ***Near HGT***, for detecting all putative intra HGT genes within a group of species (strains) 𝓢 and a reference set of organisms 𝓡:

**Procedure *Near HGT(𝓢, 𝓡, δ_SI_, δ_ρ_)***

1. for all *S*_1_, *S*_2_ ∈ 𝓢
  - for every HGT suspected gene *g*_*h*_ ∈ *S*_1_ ∩ *S*_2_ s.t. *SI*(*g*_*h*_, *S*_1_, *S*_2_) < *C*(*δ*_*SI*_)
  - let ℓ = |*g*_*h*_|
  - for *R*_1_, *R*_2_ ∈ 𝓡 s.t. *g*_*h*_ ∈ *R*_1_ ∩ *R*_2_
    – for all witness genes *g*_*w*_ ∈ *S*_1_ ∩ *S*_2_ ∩ *R*_1_ ∩ *R*_2_
      * set $\rho_{g_{h},g_{w}}\leftarrow \frac{d_{g_{h}}(r_{1},r_{2})}{d_{g_{w}}(r_{1},r_{2})}$
      * set $d^{\prime_{g_{h}}}\leftarrow \rho_{g_{h},g_{w}}d_{g_{w}}(s_{1},s_{2})$
      * set $\chi^{2}\leftarrow \frac{\ell {\left(d_{g_{h}}-d^{\prime_{g_{h}}}\right)}^{2}}{d^{\prime_{g_{h}}}(1-d^{\prime_{g_{h}}})}$
      * if the probability for *χ* ^2^ with 1 DoF is at most *δ*_*ρ*_, then mark *g*_*h*_ as putative HGT

It is important to note here that since we perform many tests for many witness genes and reference organisms, a correction for multiple hypothesis testing should be performed. We chose the standard *Bonferroni* correction, considered to be highly conservative, multiplying the bound obtained by the number of tests for a given gene.

### Simulation Study

We conducted a simulation study to assess the power of the new proposed method. Obviously, the longer the gene the greater the confidence that is obtained (more samples). Similarly, the more recent the event is (closer to the extant species) the stronger the signal. We wanted to show these effects in a simulation study.

In the study we created a random Yule [43] tree over 20 taxa that was used as the species tree. Edge lengths represent the time that passed between speciation events and distribute exponentially (see more details in supplementary text in S5 Text). We chose two pairs of organisms from the tree: *r*_1_ and *r*_2_ that were used as the reference pair, and *s*_1_ and the *s*_2_ pair between which the HGT event occurred. We evolved the witness gene *g*_*w*_ on the original tree. Then we simulated a HGT event along the path from *s*_1_ to the least common ancestor of *s*_1_ and *s*_2_, *LCA*(*s*_1_, *s*_2_). This HGT resulted in a lower ancestor to *s*_1_ and *s*_2_. Then, the HGT gene *g*_*h*_ was evolved on this tree. Both genes evolved on their respective tree, according to the Jukes-Cantor model. The four distances were taken between the resulting sequences at leaves *s*_1_, *s*_2_, *r*_1_, and *r*_2_, for both *g*_*w*_ and *g*_*h*_. We used the *χ* ^2^ test (with 1 DoF) to reject the null hypothesis (i.e., no HGT occurred). Every point in the plotted graphs is an average of 20 runs.

**Fig 3:**
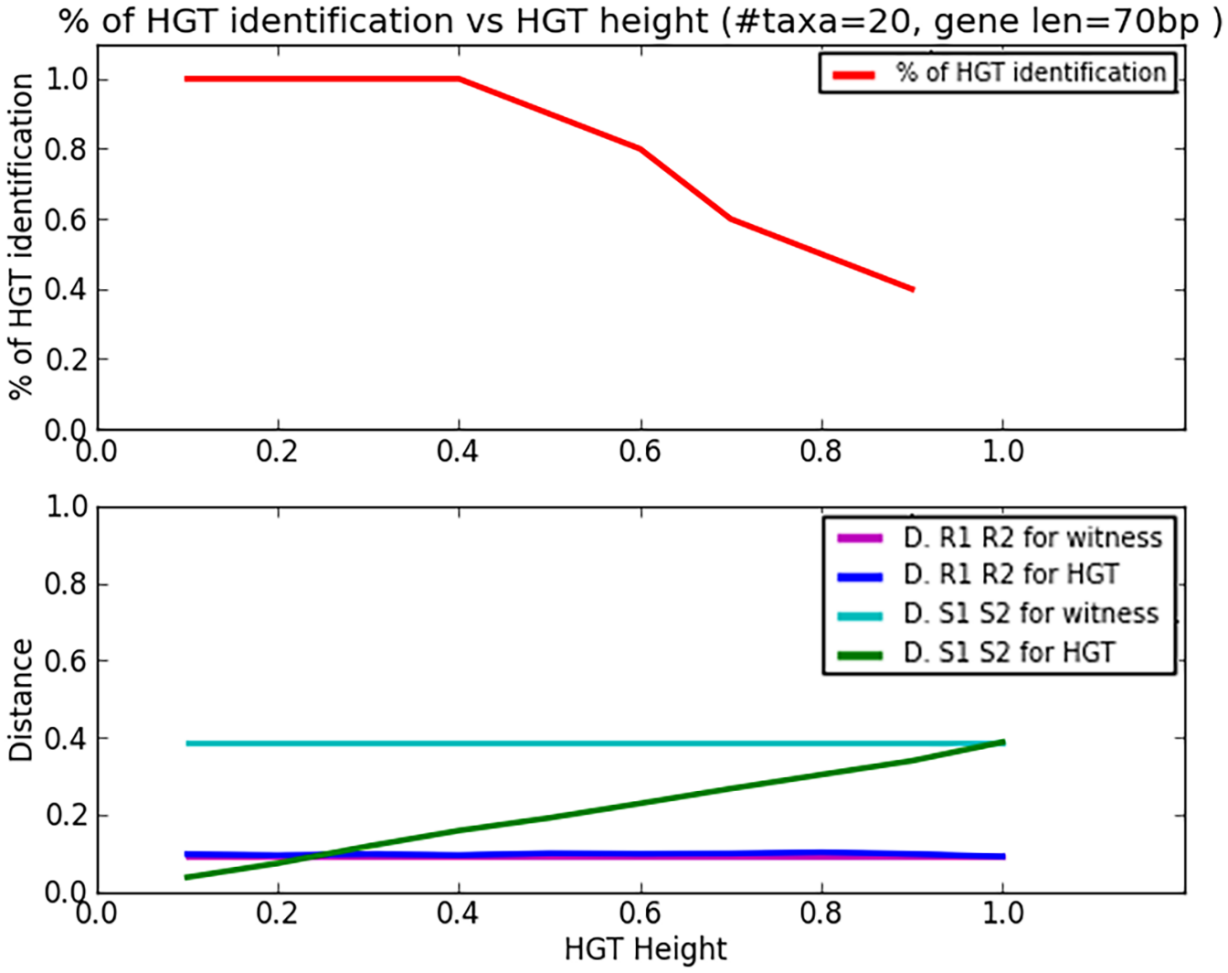
The HGT simulation study: HGT identification rate as a a function of HGT height. Gene length is 70bp.

Our first study focused on the effect of how recent the HGT event and is depicted in Fig 3 The event’s height signifies how close the event was to the leaves (i.e. recent) as a fraction of the length of the path from the leaves (*s*_1_ or *s*_2_) to the LCA, *LCA*(*s*_1_, *s*_2_), where zero implies HGT at the very leaves, and one—at the LCA. In the figure, gene length is held constant at 70bp while the HGT height varies. The top graph shows HGT identification success rate and the bottom graph shows the four distances (only one distance should change and it is the *d*_*g*_*h*__(*s*_1_, *s*_2_) when the event height changes). As can be seen the distance between the *s*_1_ and *s*_2_ according to the *g*_*h*_ grows the higher the HGT (closer to the *LCA*(*s*_1_, *s*_2_)), while all other distances are not affected, yielding fewer HGT event identifications. HGT identification is perfect until HGT height reaches 0.4 and then starts to drop. However, we still see some significant identification rate of 0.4 even at very high position of the HGT—0.9 where the sequences are almost identical, implying that under “laboratory conditions” such as these, our method is quite effective, even for short gene fragments.

In the bottom graph, we see that the distances between *r*_1_, and *r*_2_ according to *g*_*h*_ and *g*_*w*_ are the same, and hence the rates are also equal, while the distance between *s*_1_, and *s*_2_ according to *g*_*h*_ reaches its reciprocal *d*_*g*_*w*__(*s*_1_, *s*_2_) only when HGT height is one—at the LCA *LCA*(*s*_1_, *s*_2_).

**Fig 4:**
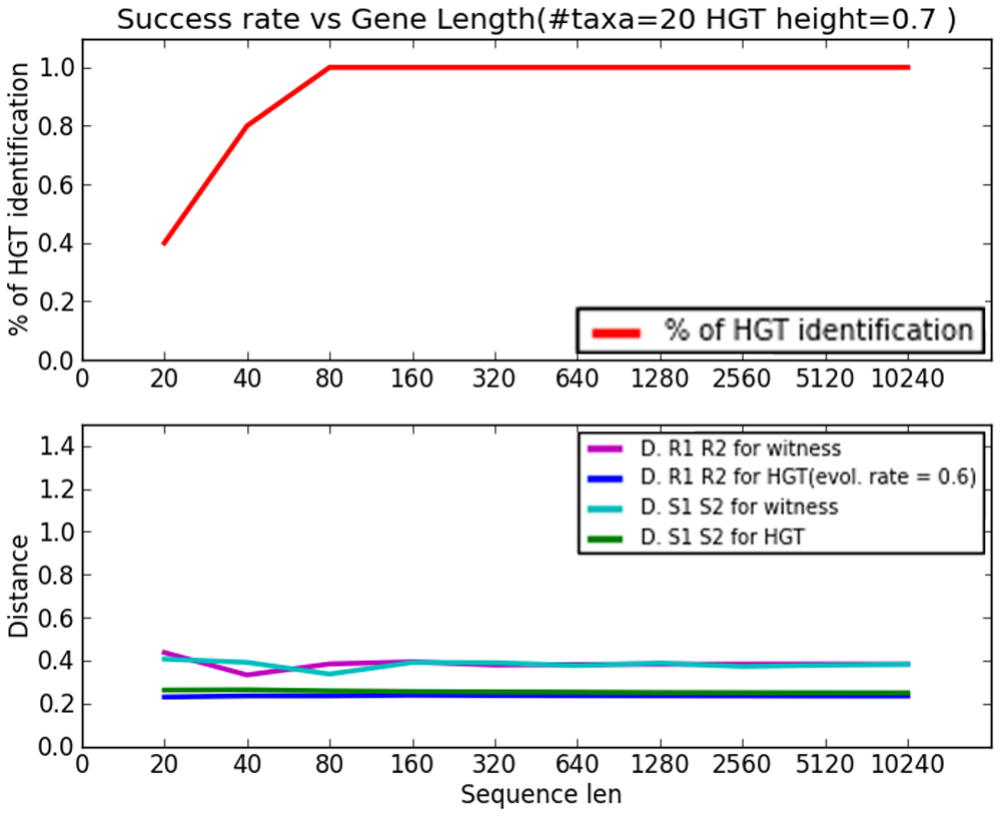
The HGT simulation study: HGT identification rate as a function of transferred fragment length. HGT event occurs at 0.7 of the height to the donor/recipient LCA. # taxa = 20 in both cases.

Our second study focused on the effect of the length of the transferred fragment and is depicted in Fig 4. Here we set the event height constant at 0.7 and varied only the length of the transferred gene. The simulation parameters remained the same as before. We see from the figure that identification starts even at quite low lengths of transferred fragments, for instance 0.4 identification rate for gene length of 20bp and achieves perfect identification (rate 1) at length 80. We note that event height 0.7 is quite challenging and a better rate is achieved for events closer to the leaves.

Also here the bottom graph in Fig 4 depicts how the four respective distances change as a result of the HGT. Unsurprisingly, distances do not change as a result of the HGT in this experiment. We see that, similarly to Fig 3, the distances *d*_*g*_*w*__(*r*_1_, *r*_2_) and *d*_*g*_*h*__(*r*_1_, *r*_2_) are the same since the two rates are the same (and of course the separating time is the same as no HGT occurred). The other two lines, representing *d*_*g*_*w*__(*s*_1_, *s*_2_) and *d*_*g*_*h*__(*s*_1_, *s*_2_), do not coincide although mutation rates are the same as HGT did occur between *s*_1_ and *s*_2_, causing the distance *d*_*g*_*h*__(*s*_1_, *s*_2_) to shrink. However, as the HGT height is constant, same is that line. It is noteworthy that the misidentification at short gene length is partly due to “incorrect” distances as a result of the stochastic process of gene evolution that we simulate.

**Fig 5:**
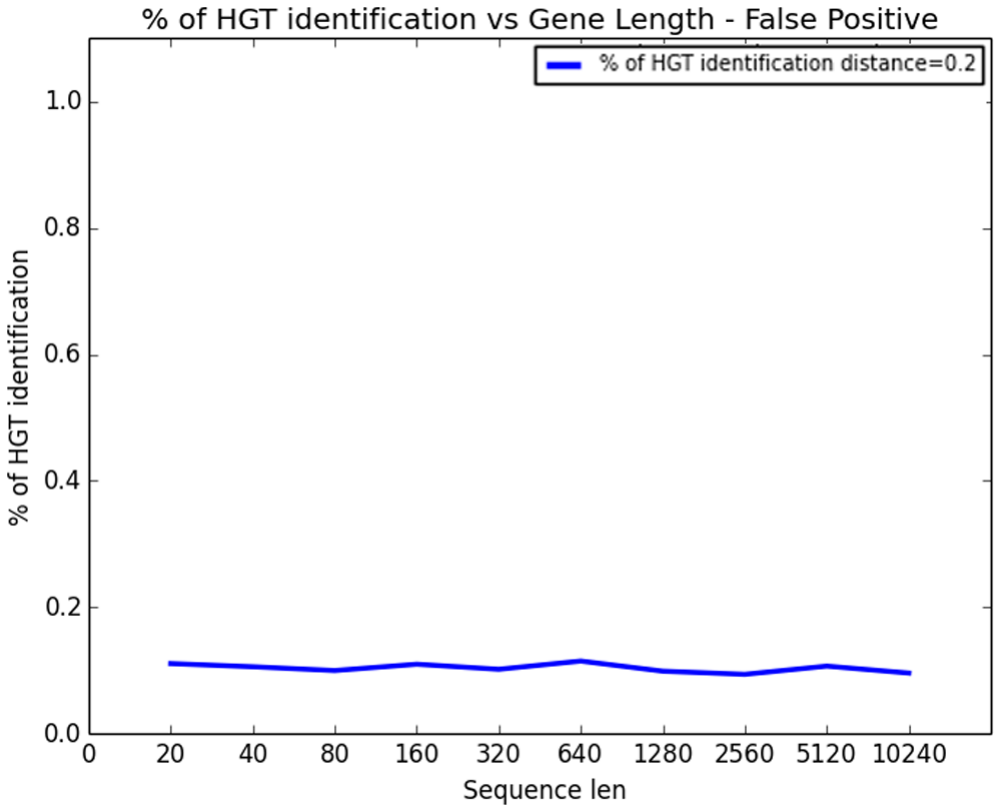
The simulation study of rate of false positive HGT detection: Rate of false positive HGT detection as a function of sequence length. Organism distance is 0.2.

**Fig 6:**
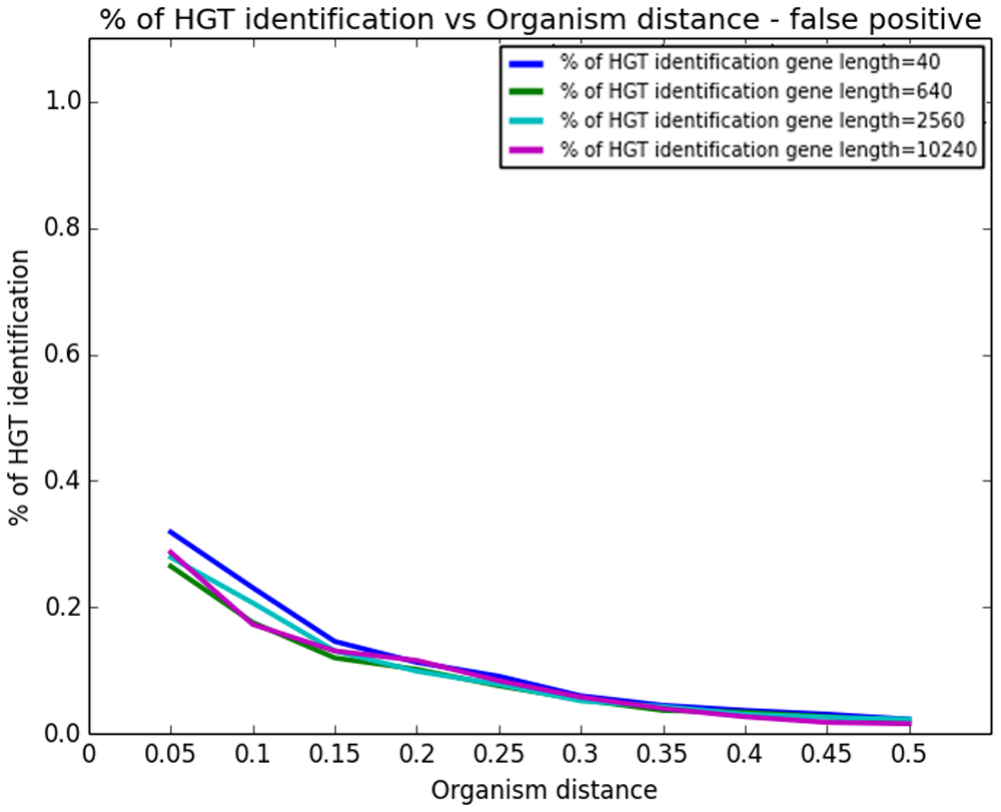
The simulation study of rate of false positive HGT detection: Rate of false positive HGT as a function of organism distance for four gene lengths—40, 640, 2.5k, and 10k bp.

Our third study addressed the question of false positive (FP) rate. As HGT is believed to be a stochastic process, our method is subjected to FP errors in the sense of alerting HGT even in the case no real HGT event took place. The first part of the study investigated the effect of sequence length on FP errors. The distance between the organisms was held fixed at 0.2 (i.e. expected number of mutations at a site 0.2). Sequence length grew exponentially from 20bp to 10k. The results are depicted in Fig 5. The second part of the study focused on the effect of the distance between the donor and recipient organisms on FP rate while the gene length is held fixed. The results appear in Fig 6. The figure shows four curves for gene length 40, 640, 2.5k, and 10k bp respectively.

As can be seen in Fig 5, as opposed to the sensitivity (or false negative) case, FP is almost entirely unaffected by sequence length. This is due to the Chi-square property that while the true parameters (distances and hence *ρ*’s) are estimated more precisely, Chi-square tends to refute the null hypothesis quicker given more data (gene length). In the contrary Fig 6 readily shows that the distance between organisms does affect FP rate. For a very short distance (closely related organisms) the signal is weak and the method is more prone to false alerts (and this holds for any sequence length, in accordance with 4.a). However, as the distance between organisms grows, the signal increases and FP rate declines.

### Analysis of Real Biological Data

*Escherichia coli* is the best-studied bacterial species, with much variation between strains, some of which are pathogenic. From an evolutionary perspective, different strains of *E. coli* exhibit highly diverse gene repertoires, reflecting much gene gain and gene loss. As such, it was of interest to look into three *E. coli* strain genomes for genes that underwent HGT and by so doing to test our method for detecting HGT between strains of the same species. Here, we used the three well-known and sequenced strains of *E. coli* studied extensively by [38]: the uropathogenic CFT073, the enterohemorrhagic strain EDL933, and the non-pathogenic laboratory K-12 strain MG1655. In general, all strains of *E. coli* underwent changes in the ancestral backbones genes at a slow rate resulting in the conserved synteny apparent across strains today. However, the remainder of these genomes is highly variable, probably reflecting numerous independent HGT events along the evolution of the different strains, and tracing back these events is challenging. Studying these three strains, one of which is an extra-intestinal pathogen, the other an intestinal pathogen and the third a non-pathogenic commensal, can shed light on the contribution of HGT to the genome evolution of pathogens.

**Fig 7:**
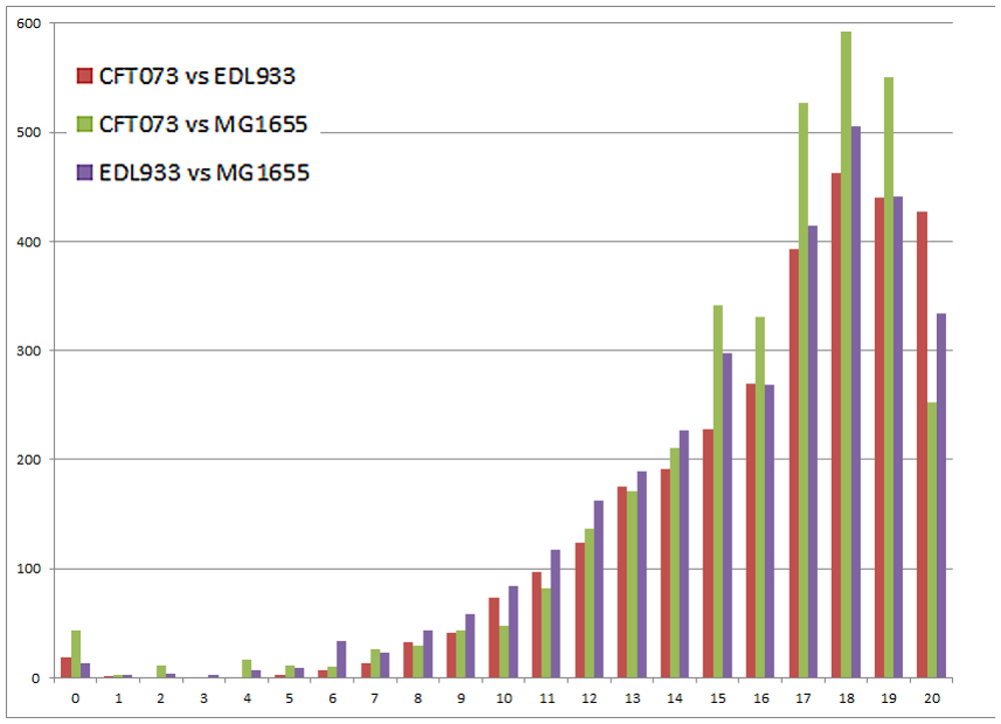
The histogram of genes’ SI values among the three pairs of *E. coli* strains. Most of the genes share the same neighborhood in all pairs, reflected by the high abundance of genes with SI = 17–20. The notable peak at SI = 0 corresponds to genes that have undergone.

**Fig 8:**
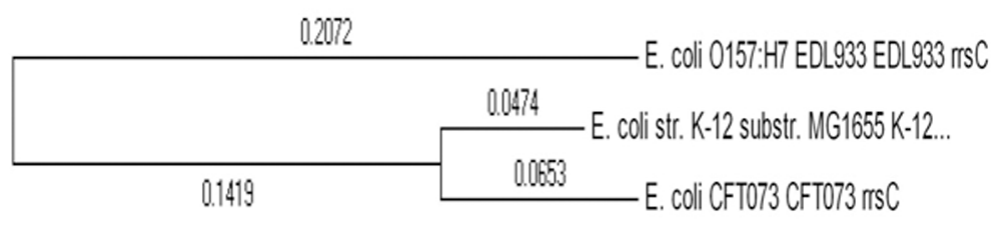
A phylogenetic tree of the three strains based on the *16S rRNA* gene. CFT073 and MG1655 are sister taxa while EDL933 is an out group.

As a first step we reconstructed the three pairwise ${\bar{SI}}_{10}(G_{i},G_{j})$ values for these three strains. The results are shown in Fig 7 and also in the table at the supplementary material (see table in S2 Table). To get some intuition on these species’ relatedness, their rate of evolution, and ancestry, we reconstructed their phylogeny based on their *16S rRNA* genes obtained from the *Ribosomal Database Project* (RDP) [44, 45]. To root the tree, a related species *Escherichia fergusonii* was used as an outgroup. The tree (without the *Escherichia Fergusonii* outgroup) appears in Fig 8. While we are aware that several other works [46, 47, 48] found different topologies over this set (i.e. different rooting), these works used different inputs and methods and also reported on conflicts between themselves. Our tree was built by the accurate maximum likelihood (ML) approach, supported by synteny data as we detail below, and also agrees with the tree obtained using seven housekeeping genes by [49]. We therefore found it sufficient for this part.

From the tree it appears that the strains CFT073 and MG1655 are sister taxa while EDL933 is an outgroup. This is in absolute agreement with our synteny-based findings, reflected in Fig 7 that we explain next. As argued before, high synteny between organisms indicates evolutionary relatedness. Therefore, between closer pairs of species we expect to find more genes with high synteny than between more distant pairs. Indeed, in Fig 7, we see greater numbers of genes with *SI* ∈ [14–19] for the CFT073- MG1655 pair (the tall green bars in the figure) than for the two other pairs (red and violet bars).

**Fig 9:**
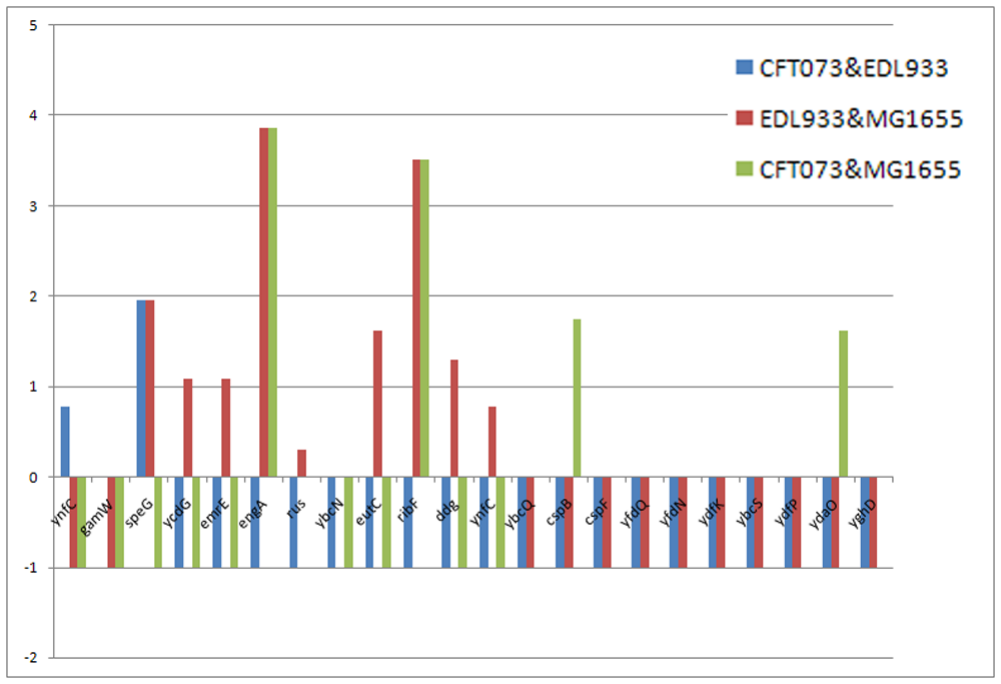
Genes with significant probability to be the product of HGT. For each significant gene, there are three bars corresponding to each pair of strains. The height of the bar represents the number of times (i.e. number of witness genes in reference species) that the gene was found with significant support (in log scale) to be derived from HGT. The value -1 indicates that this gene is not an SI-based HGT candidate between these two strains (including cases where the gene is simply not present in both strains). Zero means we did not find any significant witness for that gene.

Next we set *δ*_*SI*_ = 0.05. From the table in S2 Table at the supplementary material, it can be seen that all genes with *SI* ≤ 5 are SI-based HGT candidates. Hence we applied the algorithm ***Near HGT*** for each SI-based candidate gene. The genes found significant for having undergone HGT between each of the three pairs of strains appear in Fig 9. The height of the bars represents the (log) number of witness genes found to testify for HGT of the studied gene. The value −1 indicates that the gene was not found to be an SI-based HGT candidate in the pair of genomes.

Conspicuously, the three most prominent HGT events, detected in a pairwise genome comparisons are supported by almost exactly the same number of witness genes. This may enforce the latter finding as every gene witnessing in one pair of reference taxa, also witnessing in the other pair. Because a gene’s SI values are computed pairwise, when a gene is transferred into a recipient organism, it incurs a low SI not only between the recipient and the donor, but also between the recipient and all other organisms that contain this gene in its original (usually ancestral) location. Hence, in cases when a gene has low SI values in both pairwise comparisons, the organism in the intersection of the two pairs, is probably the recipient. That gene will have high SI values between the other two remaining genomes. Accordingly, the recipient genome is that of strain MG1655 for the genes engA and ribF, and strain EDL933 for gene speG. By our rate check in Eq (4) we can hypothesize regarding the donor organism. In the case of the *speG* gene, where the strain EDL933 appears in both pairs (that is, in the red and green bars corresponding to pairs EDL933-MG1655 and EDL933-CFT073 in Fig 9. respectively), the event could have occurred before the MG1655- CFT073 split (See the *16S rRNA* tree in Fig 8), or after the split. Both scenarios yield low SI and also unexpected rate (distance) decrease at both sister strains MG1655 and CFT073.

The case of the *engA* gene is more complicated. Here the recipient is the strain MG1655, which causes low SI with both EDL933 and CFT073. However, the rate check found this gene significant for both pairs MG1655- CFT073 and MG1655- EDL933. It cannot be that the distance to both species became shorter. Indeed a BLASTN search for the *engA* gene at the strain *MG1655* in the nr database at NCBI revealed that the closest homolog is present in *Shigella flexneri* (See BLAST output file in S1 Fig in the supplementary material). We can infer that the *engA* gene was transferred to the strain *MG1655* from an organism that was not included in the 3 strain set we investigated (in this case from a close relative of *Shigella flexneri*), causing an unexpected *increase* (as opposed to decrease) in distance as evidenced in the rate check algorithm.

In terms of nucleotide composition. these three genes have a composition that is far from striking—with G+C% of 46.34%, 53.6% and 52% for *speG*, *ribF* and *engA* respectively, similar the the *E. coli* genomic average, and confirming the hypothesis they were transferred from a recently diverged taxa. Conceivably, such similar composition is unlikely to be picked up by composition-based HGT-detection methods.

Finally, genes with only a single bar in Fig 9, may indicate existence in only that pair of organism (specifically the case of genes *ydaO* and *cspB*)

### Comparison with Other Methods

Since our approach relies on new ideas that were not explored before in the realm of HGT detection, we set to compare our approach with representative existing HGT methods.

**Fig 10:**
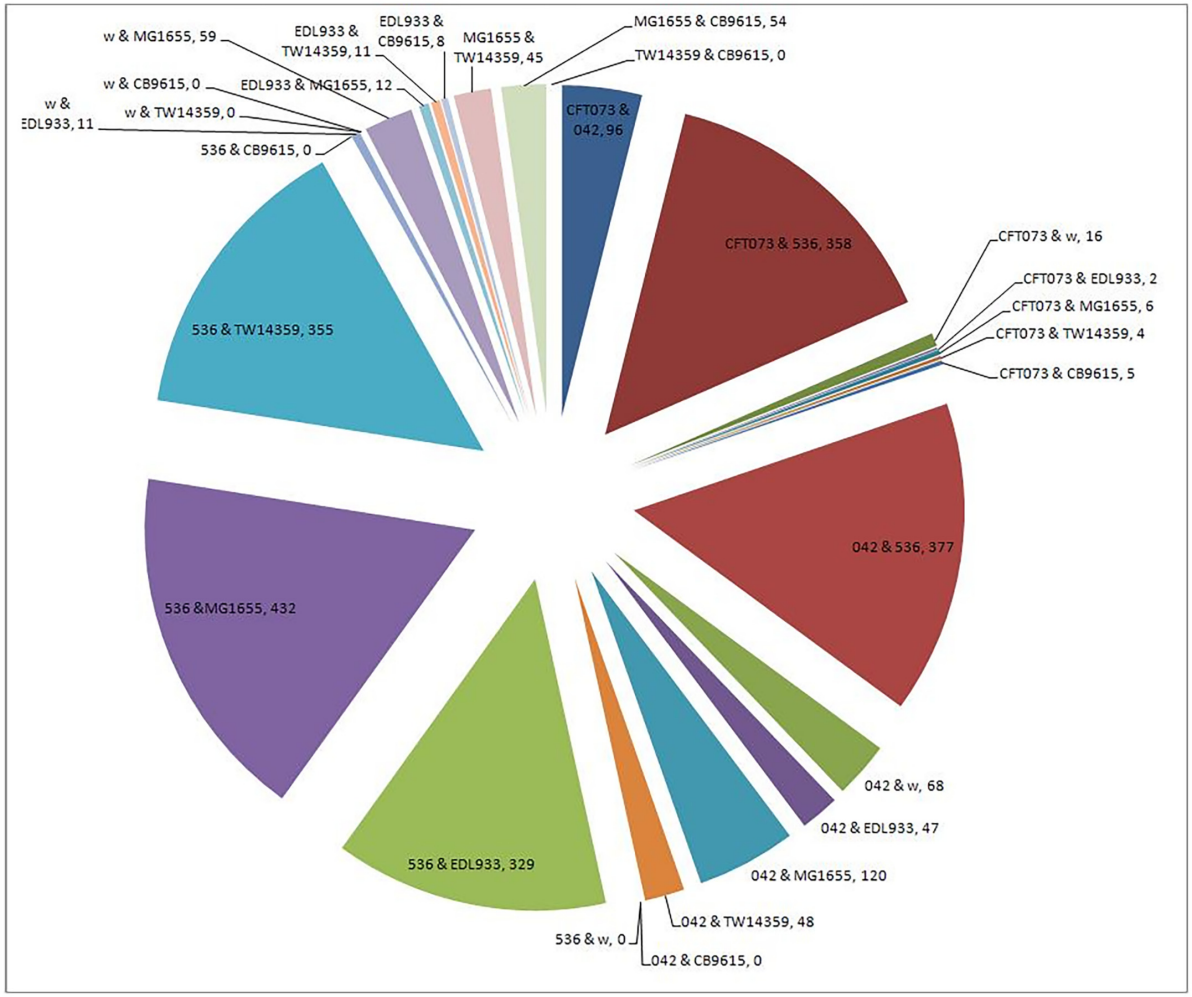
HGT events detected per strain pair. ***Near HGT*** was applied to 8 *E. coli* strains. As a result 28 pairs of strains were generated and HGT events were detected for each pair. Each piece of the pie represents two strains (e.g. 536&MG1655) and the number of identified HGT events (e.g. 432).

To substantiate the set of detected genes and allow reliable application of the phylogenetic method, we added to the strains analyzed above five more strains of *E. coli*: Enteroaggregative *E. coli* 042 (denoted 042 below), uropathogenic *E. coli* 536 (denoted 536), enterotoxigenic *E. coli* W (denoted w), enterohemorrhagic *E. coli* O157:H7 str. TW14359 (denoted TW14359) and enteropathogenic *E. coli* O55:H7 str. CB9615 (denoted CB9615). The HGT events detected when applied to the entire data (including the previously described strains), containing the eight strains, are shown in Fig 10. A list of these genes, sorted by incongruent pairs and number of witnesses is given in table S2 Table in the supplementary material.

We start with the phylogenetic approach. This approach concentrates on a specific gene and contrasts its history (phylogeny) with the species history. As was shown in the three strains analysis in Section [Analysis of Real Biological Data], a single HGT event may yield synteny incongruence between several pairs of taxa. Therefore, when working with multiple species, our approach highlights “incongruent pairs” of species that may result from one single HGT event. A closer inspection of the kind done in Section [Analysis of Real Biological Data] can reveal the source and target of the event. In this part we chose two genes that were detected as putative HGT-derived with significant support by our method but are also present in all selected strains, and additionally, perform important functions within the bacterial cell: *valS* and *speG*.

- *valS* ([50, 51]) is a Valyl-tRNA synthetase, an amino-acyl tRNA synthetase which catalyzes the attachment of valine to tRNA(Val). tRNA amino-acyl synthetases have been shown to frequently being horizontally transferred in evolution[52].
- *speG*([53]) is spermidine N1-acetyltransferase (SAT) which regulates polyamine concentration by its degradation, and is involved in the prevention of spermidine toxicity at low temperatures in *E. coli*[54]. Detoxification functions are often horizontally transferred across bacterial species [55].

We tested the *speG* and the *valS* genes for HGT within the eight *E. coli* strains using two phylogenetic methods: RIATA-HGT [39] and PhylTr [40].

### RIATA-HGT

RIATA-HGT [39] is a relaxed version of a problem of minimum-cardinality [56] which looks for the minimum number of HGT events (SPR moves, see [57]) occurring on a given species tree *S* which give rise to a given gene tree. As the problem is NP-hard, RIATA-HGT is a heuristic for that problem that runs in polynomial time but was found to provide fairly accurate results [39].

**Fig 11:**
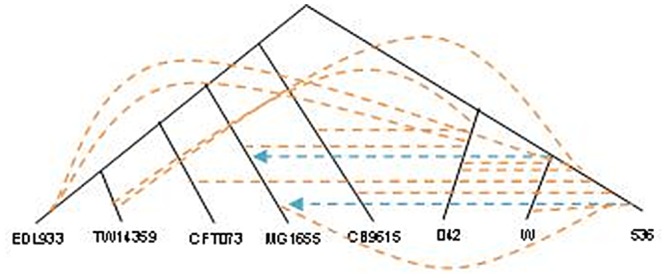
Comparison of HGT events detected by the *Near HGT* and RIATA-HGT methods for the genes *valS* and *speG*: *valS* based tree with HGT events marked by broken arrows. Blue—HGT detected by RIATA, orange—HGT detected by *Near HGT*.

**Fig 12:**
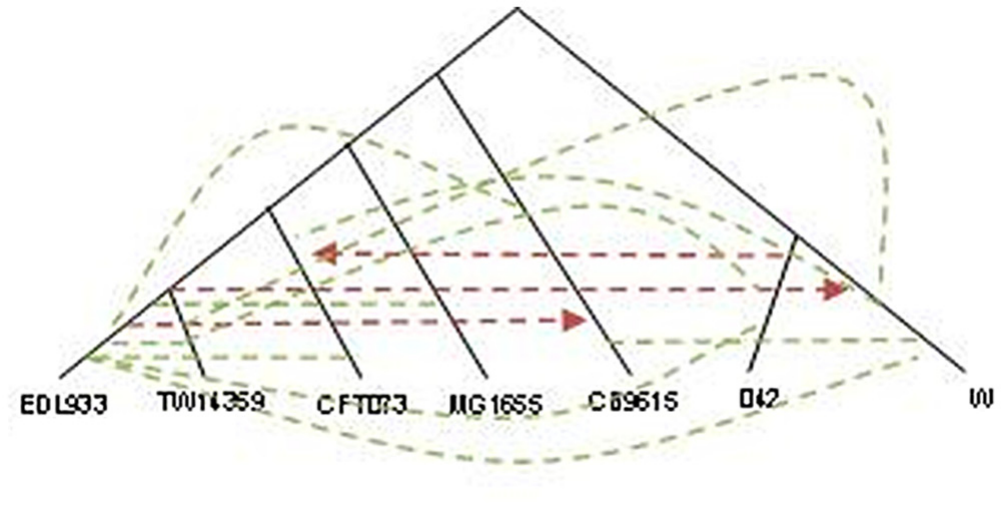
Comparison of HGT events detected by the *Near HGT* and RIATA-HGT methods for the genes *valS* and *speG*: *speG* based tree with HGT events marked by broken arrows. Red—HGT detected by RIATA, green—HGT detect by *Near HGT*.

In order to use RIATA-HGT, a species tree based on 16S rRNA gene and two gene trees based on *valS* and *speG* genes, were constructed. Next we applied RIATA-HGT over the three described trees. Examination of the RIATA-HGT results for *valS* gene (Fig 11) reveals two HGT events, while our method detected twelve incongruent pairs. While a single HGT event may yield several incongruent pairs, careful inspection of the pairs in Fig 11 gives rise to at least three events. For *speG* gene, RIATA-HGT detected three HGT events (Fig 12), largely in agreement with our incongruent pairs findings.

### PhylTR

The other phylogenetic method is PhylTR [40] that reconciles the incongruence between given species and gene trees. The chosen reconciliation is the one with a minimum number of gene duplications, losses, and lateral transfers. This method defined the DTL-scenario (Duplication-Transfer-Loss scenario), which is a formal equivalent of a reconciliation. A scenario explains how a gene tree has evolved within a species tree using duplications, HGTs, and losses. The output of this method is the trees with the most parsimonious (MP) DTL-scenarios. Applying this method (with its built-in parameter values) to our data (the 3 trees described earlier—a species tree and two gene trees) yielded the following results: *valS*—one MP tree was found with two HGT events; *speG*—nine MP trees were found with between one to three HGT events. In contrast, the *Near HGT* method was applied to all $\left(\begin{array}{c} 8\\ 2 \end{array}\right)$ pairs and found eleven incongruent pairs for *valS*, and ten incongruent pairs for *speG*. This result indicates that HGT event took place. However, further analysis as was done for the three strains (Section [Analysis of Biological Data]) for determining donors and recipients and number of events was not performed here.

### Sequence based methods

Sequence composition based methods [6, 58, 59, 60, 5] rely on the fact that certain genomic characteristics have wide variation across different bacterial species. Therefore, genes from alien origins (i.e. that were transferred horizontally) exhibit different characteristics than the typical genomic one. The characteristics that are normally investigated are the frequency of certain “words” in the genome. In order to detect such alien, atypical segments, methods work by applying a *sliding window* approach, in which the characteristics inside the window are constantly compared to those of the whole genome. When a significant difference between the window’s characteristics and those typical to the entire genome is found, it is reported as HGT suspected. However, this distinction between “alien” segments and the prevailing genome characteristics, normally “fades” throughout the time due to the phenomenon of *amelioration* [58] in which the acquired segment is adapted to the host’s genomic composition.

HGT-DB [60] is a genomic database that combines statistical parameters such as codon and amino-acid usage as well as G+C content and information about which genes deviate in these parameters from the complete prokaryotic genome. A gene is declared as HGT if it deviates by more than 1.5 standard deviations from the mean (i.e. genomic) values [22]. Additionally, there are also minimal length requirements for a transferred segment.

The HGT-DB contains only three out of the eight strains: CFT073, 536 and EDL933. In addition, out of all genes detected by SI, only *cspB* was reported as HGT in CFT073 by HGT-DB. Since segments transferred between closely related strains cannot differ too much from their host, there is no wonder that only one gene was found.

In order to apply general sequence based criteria for HGT to the genomes under study, we pursued the following general procedure [61]. For a given word length ℓ_*w*_ and a segment *S*, the *S*_ℓ_*w*__-spectrum is a $4^{\ell_{w}}$ dimensional vector holding the relative frequency of every ℓ_*w*_ long word in *S*. For a window *I* (a segment of a pre-determined length along the genome), we compute the Euclidean distance between *I*_ℓ_*w*__-spectrum and its host genome’s spectrum. This defines a distribution over the distances pertaining to the various windows along a genome. For a 0 < *δ* < 1, we say that a window *I* is **δ*-atypical* if its distance to the genome is greater than 1 − *δ* fraction of all the other distances (i.e. a *p* − *value* of *δ*). We note that for a genome with a uniform (or any other constant) distribution over the words, if window sizes are large enough, then no window will be atypical. According to the law of large numbers, every window will have very similar spectrum to the genome’s spectrum, and no window will be more distant than 1 − *δ* fraction of all the other distances.

We implemented this approach for dinucleotide [62], trinucleotide [63] and tetranucleotid content [64] (i.e. ℓ_*w*_ = 2, 3, 4). G+C content was implemented using a 2-dimensional vector holding the frequency of G+C versus A+T. Window size was set to 2000 bp and the window was moved along the genomes in steps of 10bp. We constructed the respected di-, tri-, tetra-spectra of each of the eight strains, and checked each of our suspected genes if it is 0.05-atypical. In all our tests, only one gene was found (by the tri-nucleotide experiment).

Concluding this part, comparing the *Near HGT* method with a variety of HGT detection methods, we found out that *Near HGT* extends, sometimes significantly, the other methods. The difference originates from the fact that between closely related species it is much harder to detect HGT events. On the other hand, composition-based methods facilitate detection of singleton/orfan horizontally acquired genes, as the rate check of *Near HGT* (but also phylogenetic methods) needs a genome related to the donor. For the phylogenetic methods, when reconstructing phylogenetic trees of closely related species any difference between the trees is hardly seen, even if they are not based on a conserved tree. Another source for lack of sensitivity in the phylogenetic approach, is that most of these methods are NP-hard [56] and therefore use heuristics [39] with no real guarantee on the results returned. As was shown here, Riata-HGT and PhylTR detected only a fraction of the HGT events found by *Near HGT*.

On sequence composition-based grounds, when a gene is transferred within closely related taxa, their genomeic signature is naturally highly similar, making atypical composition impossible to detect. Therefore, we observed poor sensitivity by the sequence-based methods of HGT detection, unlike the efficiency of *Near HGT*.

## Discussion

In this work we have exploited the notion of *synteny index* (SI) [27] that is useful in settings of inter-species recombination to devise a novel approach, *Near HGT*, to detect HGT between closely related taxa. We first applied it to three strains of *E. coli* and subsequently to five more (a data set of eight strains in total) and found several genes highly suspected of having undergone HGT. Our method also provides indications regarding the donor and recipient lineages by phylogenetic analysis as we demonstrated in the case of the three strains.

HGT between closely related organisms is a domain that is not covered by existing HGT methods as the signal available to these methods is very weak in this particular case. The method applies two stages of HGT detection. The first stage relies on synteny conservation between the species and discovers genes with unusual location. The second stage, exploits the key property of relative rate conservation that is maintained across species [35]. If a gene is found to exhibit both low synteny conservation with respect to another species, and also a significant deviation from the rate conservation, it is considered a validated HGT candidate.

*Near HGT* may shed light on recent gene acquisition events between related organisms, possibly only recently diverged. Identifying such events is important for the study of evolution as well as for molecular epidemiology. The latter field will benefit greatly from a more sensitive reconstruction of the emergence of virulent, often drug-resistant, strains. In the future this method will be applied to additional organisms and strains, for which genome sequences are available and integrate it with existing approaches for HGT detection so that cross validation and accurate tracing of the donors and recipients are facilitated.

## Material and Methods

### Preliminaries

**Fig 13:**
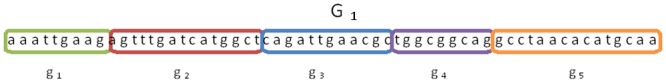
A genome is viewed as a sequence of genes while a gene is a sequence of nucleotides.

We now define our working model that will serve to locate HGT between genes. A genome is a sequence of genes (*g*_1_, *g*_2_, …, *g*_*n*_) and each gene is a sequence of DNA letters. That is, our view of a genome is at a resolution of genes, and of a gene at a resolution of nucleotides (See Fig 13.).

**Fig 14:**
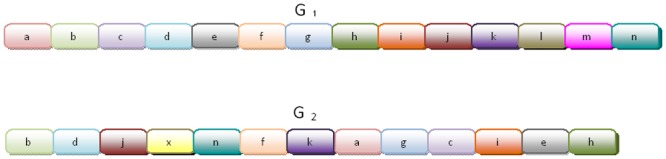
Comparing *G*_1_ with *G*_2_ for *k* = 3: *SI*(*g*, *G*_1_, *G*_2_) = 3, *SI*(*x*, *G*_1_, *G*_2_) = 0, *SI*(ℓ, *G*_1_, *G*_2_) = 0.

The *k-neighborhood* of a gene *g*_0_ in genome *G*, *N*_*k*_(*G*, *g*_0_) is the set of genes at distance at most *k* from *g*_0_ in *G* (i.e. at most *k* genes upstream or downstream). The conservation of gene order between two genomes is called *synteny*. Let *g*_0_ be a gene common to two genomes *G*_*i*_, *G*_*j*_. Then the *k* *synteny index* (*k*-SI), or just SI when it is clear from the context, of *g*_0_ in *G*_*i*_, *G*_*j*_ is the number common of genes in the *k* neighborhoods of *g*_0_ in both *G*_*i*_ and *G*_*j*_: *SI*(*g*_0_, *G*_*i*_, *G*_*j*_) = |*N*_*k*_(*G*_*i*_, *g*_0_) ∩ *N*_*k*_(*G*_*j*_, *g*_0_)|. For the sake of completeness, for *g*_0_ ∉ *G*_*i*_ ∩ *G*_*j*_, *SI*(*g*_0_, *G*_*i*_, *G*_*j*_) = 0. See Fig 14 for illustration.

Given two genomes *G*_1_, *G*_2_, and let 𝓖 be the set of genes in at least one genome, 𝓖 = *G*_1_ ∪ *G*_2_. Then the *average k-SI* between *G*_1_ and *G*_2_ is defined by

$${\bar{SI}}_{k}\left(G_{1},G_{2}\right)=\frac{1}{\left|𝒢\right|}\sum_{g\in 𝒢}\frac{SI_{k}\left(g\right)}{2k}.$$ (5)

We observe that for two identical genomes, ${\bar{SI}}_{k}(G_{1},G_{1})=1$ and for two genomes with disjoint sets of genes ${\bar{SI}}_{k}(G_{1},G_{2})=0$. The average *SI* gives us a measure of similarity between pairs of species.

A genome undergoes events of gene gain and loss in which genes are added or removed respectively. As we are focused in the core set of genes that are common to two organisms, we are not interested in the latter processes. Every gene undergoes a process of sequence evolution according to some stochastic evolutionary model [65]. The evolutionary model we consider is such that the nucleotides along a gene are identically and independently distributed (IID). The value of the nucleotide is the *state* (we sometimes use just “nucleotide” to denote its state). A *single mutation* (or *point mutation* or just a mutation for short) is the event of a nucleotide changing its value to a different one. An *evolutionary model* 𝓜 models the (stochastic) process of mutations occurring at a site as a function of *mutation rates* *α*_*i*, *j*_ modeling the rate of transitions from state *i* to *j*, and a specified time period *t*. We use the *transition* notation in the context of Markov chains and note that it has nothing to do with the type of mutation bearing the same notation (see [65] for more details). Given 𝓜, mutation rates [*α*_*i*, *j*_], and a time period *t*, the *transition probability* *p*_*i*, *j*_ from nucleotide *i* to *j* during *t* is uniquely defined by an appropriate function (determined by 𝓜). An evolutionary model 𝓜 is said to be *time reversible* if it is not possible to determine the direction of time given two states of a nucleotide, separated by a time period *t*. The *evolutionary distance* (or *mutation distance* or simply distance), *d*(*s*_1_, *s*_2_), is the number of mutations separating between two homologous sequences *s*_1_ and *s*_2_. The *Hamming distance* *h*(*s*_1_, *s*_2_) between two homologous sequences counts the number of sites with different states. Using the model 𝓜 we can convert between the two distances. These distances are usually normalized by the length of the sequences and are normally denoted by *d* and *h* respectively. As every gene exhibits a different distance between the respective sequences, we use the gene as a subscript in the distance notation, e.g. *d*_*g*_(*s*_1_, *s*_2_). In the Results section, we used the simple Jukes-Cantor [42] (JC) evolutionary model for illustration.

A *horizontal gene transfer* (HGT) is the event in which a gene of a genome, the *donor genome*, being copied and inserted at some (random) position at another genome, the *recipient genome*. Since we view the genome as a sequence of genes (see Fig 1), the new gene is always between two genes (or at the ends of the genome). colorblack By the assumption of randomness we expect the gene to have a new neighborhood.

### Data Sources

All genomes analyzed were downloaded from the NCBI microbial genomes resources [66] (http://www.ncbi.nlm.nih.gov/genomes/lproks.cgi). Appropriate 16S-rRNA genes were downloaded from the Ribosomal Database Project (RDP) [44, 45]. RDP provided two sources for trees, namely a distance based, ready made tree for selected organisms and pre-aligned sequences, based on rRNA secondary structure alignment, that are available from RDP for further independent comparative analysis (including phylogenetics). As maximum likelihood (ML) reconstruction is considered more reliable than distance based analysis, we chose to use the aligned sequences.

The names and order of genes were extracted using RefSeq annotation [67] as it provides an easy to use source of such data, especially for the well-annotated *E. coli* genomes.

The gene trees for genes *speG* and *valS* (see Figs 11 and 12) were obtained as follows. Gene sequences for the eight orthologs were extracted from the GenBank sequences and aligned using ClastalW [68].

All phylogenetic reconstruction (including the *16S rRNA* was done using ML reconstruction under the GTR + Gamma evolutionary model (designed for sequences with significant between-site rate heterogeneity). We used the PhyML software [69] to build tree from the aligned sequences (with the parameters indicated above).

## Supporting Information

S1 Datasets example procedure for detection of horizontal gene transfer between given strains: *nearHGT*\*WasThereHGT*\*WasThereHGT*.*py*, *nearHGT*\*WasThereHGT*\*SampleSeq*.*py*, *nearHGT*\*WasThereHGT*\*BuildGenF*.*py*. (PY)

S2 Datasets the procedure used for the simulation study presented in the article: *nearHGT*\*Detection*-*Of*-*HGT*-*simulation*\*BuildGenF*.*py*, *nearHGT*\*Detection*-*Of*-*HGTsimulation*\*sim*-*detc*-*HGT*.*py*, *nearHGT*\*Detection*-*Of*-*HGT*-*simulation*\*tree*.*py*. (PY)

S1 Fig *engA-blast-search.png*: BLAST output file. (PNG)

S1 Table *organism.xls*: List of strains used in the biological analysis and their NCBI ID. (XLS)

S2 Table *suppl*\*SI*_*strains*_*table*.*xlsx*: SI strain table for every analyzed pair of strains. (XLSX)

S1 Text *nearHGT*\*WasThereHGT*\*Readme*.*txt*—file that explains how to run the example program, *WasThereHGT.py* for detecting HGT. (TXT)

S2 Text *nearHGT*\*WasThereHGT*\*genesec*.*txt* sample of 8 gene sequences. Length of each sequence: 70 nucleotides
(TXT)

S3 Text *strains-16s.fasta*: alignment of 16S of the analyzed strains. (FASTA)

S4 Text *strains-16s.nwk*: 16s tree of the analyzed starins in newick format. (NWK)

S5 Text *supplText*_*Dec*-25-2014.*pdf*: Text that describes the simulation study algorithm in detail. (PDF)
