# Supplementary figures and images for: Detecting Horizontal Gene Transfer between Closely Related Taxa
Source: PLoS Comput Biol. 2015 Oct 6;11(10):e1004408. doi: 10.1371/journal.pcbi.1004408 (PMC4595140; doi:10.1371/journal.pcbi.1004408)

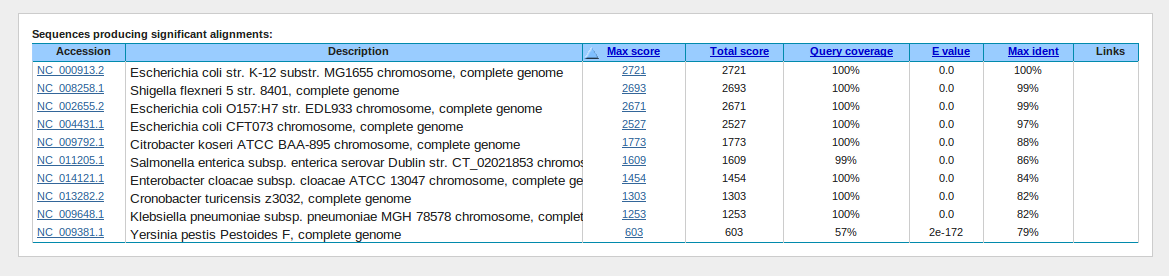

Supplement: S1 Fig — (PNG) [file pcbi.1004408.s003.png]
