# Supplementary material for: Detecting Horizontal Gene Transfer between Closely Related Taxa
Source: PLoS Comput Biol. 2015 Oct 6;11(10):e1004408. doi: 10.1371/journal.pcbi.1004408 (PMC4595140; doi:10.1371/journal.pcbi.1004408)
Supplement: S5 Text — (PDF) [file pcbi.1004408.s010.pdf]

supplementary Material

We here give a detailed description of technical parts that were left outside of the main paper body.

1 Detection of HGT event based on Constant Relative Mutability (CRM) hypothesis

We have simulated several HGT events on a given species tree. The simulation consisted of three main simulation runs, with different parameters:

1. Simulation of HGT events that occurred at different time distances on the species tree, for a given sequence length
2. Simulation of HGT events that occurred at a specific time distance on the species tree, on genes with different sequence lengths
3. Simulation of false positive detection of HGT event

Description of general flow of simulation: For the simulations we have generated a random species tree with twenty organisms. This tree was used as a reference tree. Then, we chose two pairs of organisms from the tree: First pair of organisms - R_1 and R_2, were used as reference organisms, and second pair of organisms - H_1 and H_2 were chosen to have undergone an HGT event, as a *donor* and *recipient.* We have simulated few different HGT events between H_1 and H_2. After every HGT event, based on a mutability ratio between H_1 and H_2 and reference pair of species R_1 and R_2, we have calculated the mutability ratio of the *" suspected gene"* of the species H_1 and H_2 (who went through HGT event), to the mutability ratio of a *witness* gene sequence from the reference tree.
Than we have compared the distance between H_1 and H_2 after the HGT event, to their expected distance (calculated using the CRM ratio), and used Chi Square test to determine whether an HGT event occurred with a certain degree of confidence.

1.1 HGT Events at Varied Event's Height and Constant Fragment Length

The first simulation run, simulated HGT events that took place at several time distances on a given sequence length. This run had several steps:

Creating a species tree with 20 species: We have generated a random Yule tree with 20 species. This tree was used as our reference tree.(See Figure 1)

Building a gene sequence with a given length and evolving it on the reference tree: we have built a sequence of a *witness gene* with 70 nucleotides. This sequence was used as the ancestral genome on our reference species tree. In the process of evolving the *witness gene* on the reference species tree, we started at the root level of the species tree and evolved it on the tree. The edge length between two nodes represented the time passed between two speciation event, and determined the amount of mutation that took place: mutation events were generated on edge with probability proportional to the time between speciation(edge length).

choosing two pairs of organisms from the tree: Once we got the evolved witness gene for all species on the tree, we chose two pairs of organisms from the tree: R_1 and R_2 that were used as reference pair, and H_1 and H_2 that were also used as reference on the reference tree and were chosen to undergone an HGT event on next step of the simulation.

Calculating *Constant Relative Mutability (CRM)ratio:* In order to be able to identify an HGT event we relied on the fact that, every orthologous genes that belong to two different species, will keep a *constant ratio of mutation rate*, at any point in time, unless they have gone through extraordinary mutation event like HGT. Based on this, we calculated the mutation ratio for the witness gene, for the two pairs of organisms that we chose: R_1 and R_2 that were used as reference pair and H_1 and H_2.

Simulating an HGT Event: We have simulated an HGT event between the chosen organisms - H_1 and H_2 performing the following steps: We calculated the distance between the organism pair H_1 and H_2 on the reference tree (this equals twice the height of their Least Common Ancestor (LCA)). Than we located a new node on the tree at height of 0.9 of the LCA height and connected to it the clade of H_1. This new tree structure (HGT

tree), with the new distance between H_1 and H_2, used for simulating the HGT event, since it included the same set of twenty organisms. Evolving a gene on the new tree structure resulted with closer distance between H_1 and H_2. This step was performed nine times. Every time we simulated HGT event in different distance from LCA (we refer to LCA between H_1 and H_2 on the reference tree), the distance ranged from distance of 0.9 *(LCA of H_1 and H_2) to 0.1 *(LCA of H_1 and H_2) (See sample HGT Tree in Figure 2).

Evolving gene on the HGT tree: We have built a gene sequence of 70 nucleotides and used it as ancestral gene, and evolved it on the HGT tree. This was done using same procedure that was used to evolve the *witness gene* sequence on the reference tree as described above. Since the gene was evolved on the HGT tree we created, we referred to it as *suspected gene.*

Calculating the distances on HGT tree: We wanted to detect the HGT event that was simulated. First we have calculated the *suspected gene* distances between the organisms R_1 and R_2 and H_1 and H_2 on the HGT tree.

Detecting an HGT event based on distances and CRM hypothesis: Using CRM hypothesis and the distance between R_1 and R_2 on the HGT tree, we calculated the expected distance between the two species H_1 and H_2 on the HGT tree for the *suspected gene.* Once we got the expected distance between H_1 and H_2 and the observed distance between H_1 and H_2, we used χ^2 significance test between the observed and expected values to decide whether the *suspected gene* has undergone HGT. Our null hypothesis is that for the *suspected gene*, an HGT event did not occur between H_1 and H_2. We refute the null hypothesis, that is, decree HGT, if the χ^2 probability with one degree of freedom is below the χ^2 test threshold value for significance level of 0.05.

The last three steps have been repeated several times. Every point in the graph represents the fraction of times the event is detected.

1.2 HGT at a Constant Height and Varying Sequence Length

The second simulation run, simulated HGT events that took place at a specific time distance on the species tree, on different lengths of gene sequences. The steps of this simulation were similar to the steps included in the first simulation run with slight differences:

choosing two pairs of organisms from the tree: We chose two pairs of organisms from the tree: R_1 and R_2 that were used as reference pair and H_1 and H_2 pair that were also used as reference on the reference tree and were chosen to undergone an HGT event on next step of the simulation.

Building a gene sequence for different sequence lengths and evolving it on the reference tree: We have built ten gene sequences that were used later as *witness genes* with ten different lengths. The first sequence was a short sequence of 20 nucleotides, and each following sequence length was double of its previous: i.e the second sequence length was of 40 nucleotides, the third was of 80 nucleotide and so on. Each generated sequences was used as ancestral genome and was evolved on the reference species tree to create ten *witness genes* in 10 different length for chosen organisms R_1 and R_2 and H_1 and H_2 on the reference tree. In the process of evolving the *witness gene*s on the reference specie tree, we started at the root level of the species tree and evolved it on the tree. The edge length between two nodes represented the time passed between two speciation event, and determined the amount of mutation that took place: mutation events were generated on edge with probability proportional to the time between speciation(edge length).

Simulating an HGT event: We have simulated an HGT event between the chosen organisms - H_1 and H_2 performing the following steps: We calculated the distance between the organism pair H_1 and H_2 on the reference tree. We have locate a new node on the tree at height 0.7*(height of reference tree), and reconnected H_1 and H_2 to the new node. We referred to the new created tree as HGT tree.

Evolving genes on the HGT tree: We have used the ten sequences with different length that we have built above, as ancestral sequences and evolved them on the new HGT tree. This was done using same procedure that was used to evolve the *witness gene* sequence on the reference tree as described above. Since the genes were evolved on the HGT tree we created, we referred to each one of them as *suspected gene*. Than (as we described in the following paragraph) for every *suspected gene* with every length, we tried to detect whether an HGT event took place using gene distance and CRM hypothesis.

Calculating the distances on HGT tree: We wanted to detect the HGT event that was simulated. For each gene sequence (after performing the HGT event), we calculated the *suspected gene* distances between the organisms R_1 and R_2 and H_1 and H_2 on the HGT tree.

Detecting an HGT event based on distances and CRM hypothesis: Using CRM hypothesis and the distance between R_1 and R_2 on the HGT tree, we calculated the expected distance between the two species H_1 and H_2 on HGT tree for the *suspected gene*. Once we got the expected distance between H_1 and H_2 and the observed distance between H_1 and H_2, we used χ^2 significance test between observed and expected value to decide whether the *suspected gene* has undergone HGT event. Our null hypothesis was that for the *suspected gene*, an HGT event occurred between H_1 and H_2. We refuted the null hypothesis, if the χ^2 probability with one degree of freedom was below the χ^2 test threshold value for significance level of 0.05.

The last three steps were repeated several times. We counted the number of times we refuted the null hypothesis and at the end of the process, we have a plotted the graph showing for every sequence length, the percentage of times we detected an HGT event.

1.3 False Positive detection of HGT event

We wanted to calculate the rate of false positive detection of HGT event when no HGT event took place. We chose two pairs of organisms R_1 and R_2 that were used as reference pair and S_1 and S_2 pair that were used as reference on the *witness gene* tree and were suspected to undergone HGT event on the *suspected gene* tree. Using CRM hypothesis and distance between R_1 and R_2 and S_1 and S_2 on the *witness gene* tree, we calculated the expected distance between S_1 and S_2 on the *suspected gene* tree. Than we have compared the distance between S_1 and S_2 pair after evolving it on the *suspected gene* tree, to their expected distance, and used Chi Square test to determine whether an HGT event occurred with a certain degree of confidence.

False positive detection of HGT event at a constant distance and varying sequence length: We wanted to check the impact of sequence length on the rate of false positive detection of HGT.
We have determined a distance between the chosen organisms and calculated

its mutation probability. The probability was proportional to the time between speciation (distance between the species) and was calculated based on Jukes-Cantor evolutionary model. Based on this model, every position in the genome had undergone a mutation event with probability $(3/4)(1-e^{-(4/3)\ell})$, where ℓ is the distance length between the chosen organisms. In our procedure, for specific genome sequence length, for each nucleotide position in the sequence, we tossed a random number between 0 to 1: If the number was less than or equal to the probability, we counted it as a mutation. The number of the mutations we got for the genome sequence, represented the hamming distance between the two species. Based on CRM hypothesis we calculated the ratio between of mutation rate of the two pairs of species for the *suspected gene*. Since we chose same mutation rate for the two pairs, the ratio was expected to be equal to one. We compared the calculated mutation ratio of the two pairs of organisms to the expected ratio and used Chi Square test to determine whether HGT event occurred with 0.95 level of confidence. We repeated this procedure 1000 times for several genome sequence lengths. We started from a short sequence of 20 nucleotides and each following sequence length was double of its previous: i.e the second sequence length was of 40 nucleotides, the third was of 80 nucleotide and so on.

False positive rate of HGT Detection at a Constant Sequence Length and Varying Distance: We wanted to check the impact of the distance between organisms on the rate of false positive detection of HGT. We chose two pairs of organisms R_1 and R_2 that were used as reference pair and S_1 and S_2 pair that were used as reference on the *witness gene* tree and were suspected to undergone HGT event on the *suspected gene* tree. We started to run our main procedure for a distance of 0.05 between every pair of species and ran several iteration while in every iteration the distance between the species was increased in 0.05: i.e. in the second iteration the distance between every pair of species was 0.1 in the third iteration the distance between every pair of species was 0.15 and so on. For every distance iteration, we calculated the mutation probability. Similar to the previous procedure, here as well, the probability was proportional to the time between speciation (distance between the species) and was calculated based on Jukes-Cantor evolutionary model. Based on this model, every position in the genome had undergone a mutation event with probability $(3/4)(1-e^{-(4/3)\ell})$, where ℓ is the distance length between the chosen organisms. For the specific genome sequence length, for each nucleotide position in the sequence, we tossed a random number between 0 to 1: If the number

was less than or equal to the probability, we counted it as a mutation. The number of the mutations we got for the genome sequence, represented the hamming distance between the two species. Based on CRM hypothesis we calculated the ratio between of mutation rate of the two pairs of species for the *suspected gene*. Since we chose same mutation rate for the two pairs, the ratio was expected to be equal to one. We compared the calculated mutation ratio of the two pairs of organisms to the expected ratio and used Chi Square test to determine whether and HGT event occurred with 0.95 level of confidence.

1.4 Yule tree generation

A Yule process advances recursively and builds a tree while advancing. At every recursion step the node with the earliest time point is chosen and processed. At node's processing two *edge lengths* are tossed randomly from a predefined distribution. The values received in the toss, are used be the edge lengths to the two children of the chosen node and their time points will be their ancestor's time plus their edge lengths. When the set of yet unprocessed nodes contains n nodes, the recursion terminates and all nodes are assigned with the time of the earliest unprocessed node. Such a procedure generates an ultrametric tree (or *molecular clock* tree) in which the distance (path length) from the root to any leaf, is the same.
Edge length describes a birth Poisson process that distribute exponentially with rate ℓ. p represents the probability of an event occurring during this time period. The procedure receives p as a parameter and transforms it to the corresponding time period, that we denote by *length* $\ell = -log(1-p)$. Next, edge length leading node v is drwan randomly end exponentially, $l_v \sim Exp(\ell)$. Hence we obtain that edge lengths for our tree are exponentially distributed with length $\ell = -log(1-p)$.

1.5 Simulating Genome Evolution

To generate genomes, i.e. gene sequences, according to the species tree we first define the ancestral root genome (usually 1,2,3..N for simplicity) and then propagate it down along the species tree. Hence, given any ancestral genome we obtain its two children according to their edge lengths with the following procedure - every gene in the child node v's genome undergoes an *event* with probability $(3/4)(1-e^{-(4/3)\ell_v})$. The probability was proportional to the time between speciation(edge length) and was calculated on Jukes-

Cantor evolutionary model, where ℓ_v is the length of the node entering v (i.e. the edge from v's ancestor to v). In our procedure, for each nucleotide position in the sequence, we tossed a random number between 0 to 1. If the number was less than or equal to the probability, we performed a mutation, by randomly choosing one of four nucleotides ('A', 'G', 'C' or 'T'), and replaced this position with the chosen nucleotide.

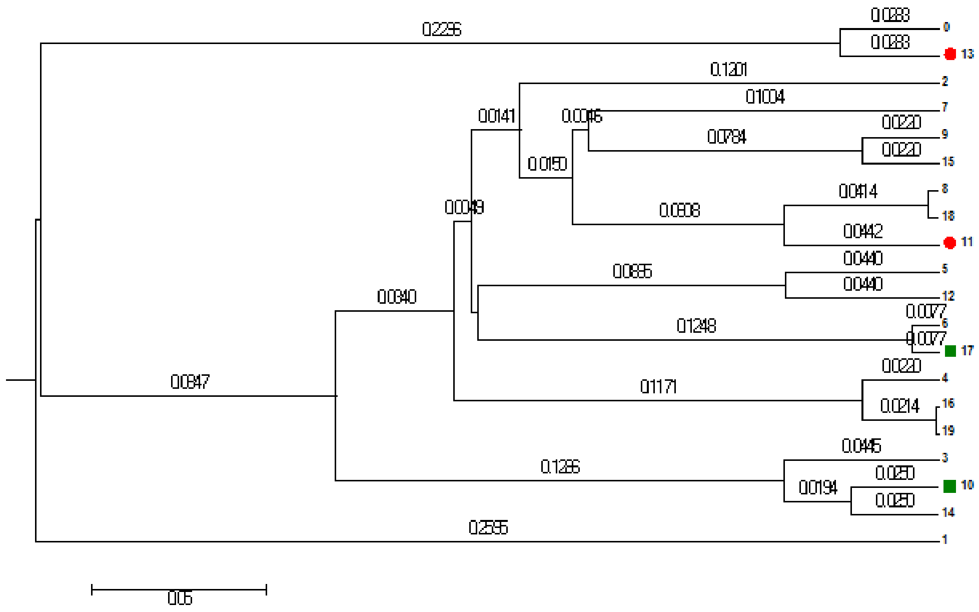

Figure 1: Refernce Yule Tree - The green bullets mark the reference organisms R_1 and R_2 (leaves 10 and 17). The red bullets mark the organisms H_1 and H_2 that were chosen to go under HGT event (leaves 11 and 13). (see next figure).

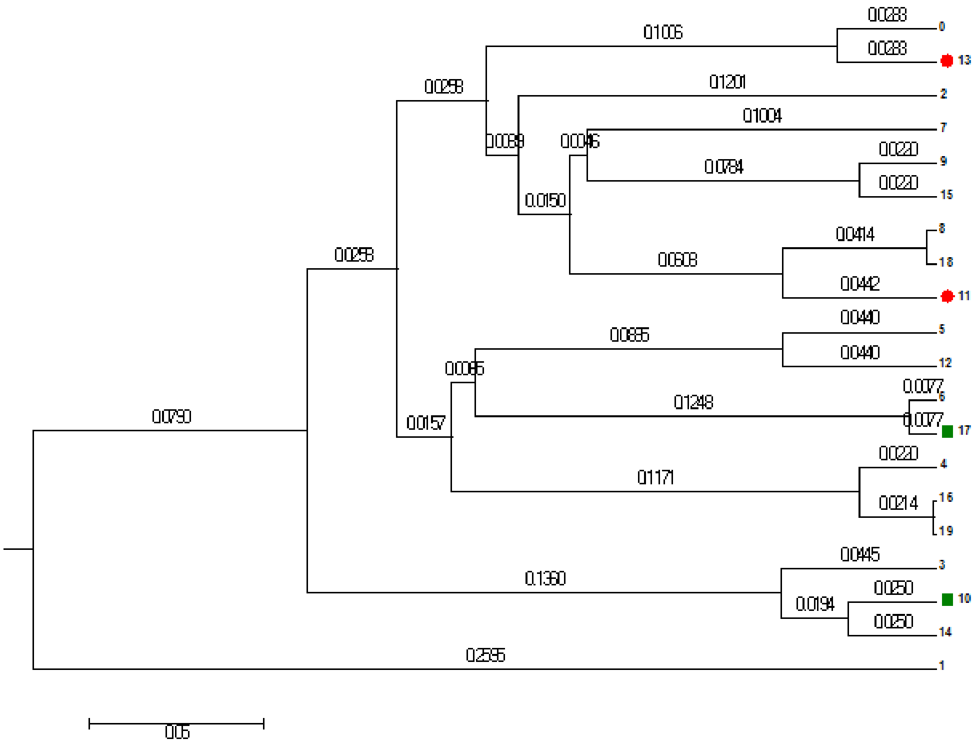

Figure 2: Sample HGT Tree - After performing and HGT event between organisms H_1 and H_2 (leaves 11 and 13). The organisms that undergone the HGT event were reconnected at height 0.5 of the reference tree. The green bullets mark the reference organisms R_1 and R_2 (organisms 10 and 17) their distance (comparing to their distance on the reference tree) was not changed. The red bullets mark the organisms H_1 and H_2 that went under HGT event (organisms 11 and 13).
